# The gut-brain-vascular axis: converging neuroinflammatory and hypertension-mediated pathways from gut dysbiosis to cognitive decline

**DOI:** 10.3389/fmed.2026.1928026

**Published:** 2026-10-19

**Authors:** Shaurya Mehta, Pranav Loomba, Hamoudi Musselmani, Jaret Ly, Sami Nguyen, Alex Niemann, Sree Kolli, David Sant

**Affiliations:** 1 University of Utah Health, Salt Lake City, UT, United States; 2 Department of Biomedical Sciences, Noorda College of Osteopathic Medicine, Provo, UT, United States; 3 California University of Science and Medicine, Colton, CA, United States; 4 Saint Louis University School of Medicine, Saint Louis, MO, United States

**Keywords:** cognitive decline, dementia, gut microbiome, gut-brain axis, hypertension, neuroinflammation

## Abstract

Hypertension and cognitive decline are major public health burdens, and both have been associated with gut microbiome dysbiosis. We propose the gut-brain-vascular axis as a testable synthesis in which dysbiosis may contribute to cognitive decline through two converging routes: direct neuroinflammatory signaling and hypertension-mediated cerebrovascular injury. In the proposed direct route, intestinal barrier dysfunction, lipopolysaccharide translocation, altered microbial metabolites, microglial activation, and vagal signaling may promote neuroinflammation. In the indirect route, dysbiosis-associated metabolites, immune activation, autonomic imbalance, and endothelial injury may contribute to hypertension, which is linked to cerebral small vessel disease, impaired cerebral autoregulation, neurovascular uncoupling, and blood-brain barrier dysfunction. The component relationships are supported to different degrees, but the complete temporal pathway and an interaction between the two routes have not been established in humans. This narrative review synthesizes gut-hypertension, gut-cognitive, cerebrovascular, shared-mechanism, and therapeutic evidence and identifies the study designs needed to test the framework. Longitudinal cohorts and trials that measure microbiome, blood pressure, cerebrovascular, and cognitive outcomes together are required to determine whether the proposed routes occur independently, sequentially, or interactively.

## 1 Introduction

Cognitive decline and dementia are major public health burdens. Approximately 55 million people worldwide live with dementia, a number projected to reach 139 million by 2050. Hypertension, affecting roughly 1.28 billion adults globally, remains the single most important modifiable risk factor for stroke and vascular cognitive impairment (VCI), and it contributes substantially to Alzheimer's disease (AD) risk. The Atherosclerosis Risk in Communities cohort linked midlife hypertension with greater 20-year cognitive decline across multiple cognitive domains (1). Blood pressure control, particularly when initiated in midlife, attenuates, but does not eliminate dementia risk (2).

Simultaneously, a convergent line of evidence has emerged linking gut microbiome dysbiosis to both hypertension and cognitive decline. The gut harbors approximately 38 trillion microorganisms (3) that collectively produce thousands of bioactive metabolites, modulate host immune function, regulate autonomic tone, and maintain barrier integrity. Disruption of this ecosystem, clinically known as gut dysbiosis, has been documented in patients with essential hypertension, coronary artery disease, chronic kidney disease, AD, and VCI. Gut microbiota alterations represent an important upstream contributor to multiple chronic noncommunicable diseases (4).

Prior reviews have described dietary salt-microbiome pathways to vascular and cognitive dysfunction and have examined microbiota as a modifier of hypertension-mediated organ damage (5, 6). The present review builds on that work by organizing the evidence around two concurrent but unequally supported routes from gut dysbiosis to cognitive decline: a direct neuroinflammatory route and an indirect route in which hypertension contributes to cerebrovascular injury. Its intended contribution is a testable synthesis of these literatures, rather than evidence that the complete pathway or an interaction between the two routes has already been established.

This review proposes the gut-brain-vascular axis as a conceptual model comprising three nodes: (1) gut dysbiosis, (2) hypertension-mediated cerebrovascular injury, and (3) direct neuroinflammatory brain injury. Throughout this review, “vascular” refers primarily to cerebrovascular injury downstream of hypertension; it is not used as a substitute for the broader spectrum of vascular disease.

The emphasis on cerebrovascular consequences reflects the proposed role of hypertension as an intermediate between a gastrointestinal exposure and a cognitive outcome. The framework remains anchored in gastroenterology because its upstream variables are microbial ecology, intestinal barrier function, mucosal immune signaling, and microbial metabolites. Cerebrovascular physiology is reviewed only to assess whether and where those gut-derived exposures could plausibly modify hypertension-related brain injury.

### 1.1 Literature identification and synthesis

This narrative review was informed by structured PubMed searches conducted on 18 May 2026 across six domains: gut microbiome and hypertension; gut microbiome and cognitive decline; hypertension and cognitive decline or cerebral small vessel disease; studies spanning microbiome, blood pressure, and cognition; shared molecular pathways; and microbiome associations with blood-brain barrier or small-vessel outcomes. We prioritized human longitudinal and interventional studies, systematic reviews, Mendelian randomization studies, and mechanistic experiments that informed a specific link in the proposed framework. Cross-sectional, animal, and *in vitro* findings were retained when direct human evidence was unavailable and are identified by design in the text. This was a narrative synthesis rather than a systematic review; no pooled effect estimate or formal risk-of-bias synthesis was performed. The description of the search and the explicit presentation of evidence levels address core quality domains for narrative reviews (72).

## 2 The gut microbiome in hypertension

### 2.1 Dysbiotic signatures in hypertension

The earliest systematic evidence linking gut dysbiosis to hypertension emerged from metagenomic profiling of hypertensive patients compared to normotensive controls. Li and colleagues (7) demonstrated that hypertensive patients harbor a distinct gut microbiome characterized by reduced microbial diversity, decreased abundance of short-chain-fatty-acid-producing genera (*Roseburia*, *Faecalibacterium prausnitzii*, *Butyrivibrio*), and enrichment of pro-inflammatory taxa, particularly *Prevotella* and *Klebsiella*. Critically, fecal microbiota transplantation (FMT) from hypertensive humans into germ-free rats elevated recipient blood pressure, providing causal evidence that the dysbiotic microbiome promotes hypertension rather than merely accompanying it (7).

Wilck and colleagues (8) established a specific mechanistic link to immune activation in mouse models. High-salt diets depleted intestinal *Lactobacillus murinus*, which promoted expansion of pro-inflammatory T helper 17 cells and elevated blood pressure. Probiotic supplementation with *L. murinus* reversed T helper 17 cell expansion and blood-pressure elevation.

The American Heart Association's 2025 Science Advisory (9) formally recognized gut microbiome dysbiosis as an emerging modifiable contributor to hypertension, endorsing it as a legitimate therapeutic target. Dysbiotic signatures consistently identified across studies include: decreased *Akkermansia muciniphila* (barrier-protective), *Bifidobacterium*, and *Lactobacillus* species; and increased *Proteobacteria*, *Helicobacter pylori*, and lipopolysaccharide-producing gram-negative taxa (10, 11).

These observational signatures do not establish directionality. Hypertension may itself alter diet, physical activity, gastrointestinal transit, medication exposure, and healthcare use, each of which can affect microbiome composition. Age, adiposity, diabetes, renal function, socioeconomic factors, and polypharmacy may also confound reported associations. Host variables can create apparently disease-specific microbiome signatures, and medication effects can be mistaken for disease effects (78, 80). Human dysbiosis signatures should therefore be interpreted as disease-associated patterns unless temporality or an intervention supports a directional claim.

### 2.2 Microbial metabolite mechanisms

Trimethylamine N-oxide (TMAO). Dietary choline, lecithin, and carnitine are metabolized by gut bacteria to trimethylamine, which is absorbed and converted by hepatic flavin-containing monooxygenase 3 (FMO3) to TMAO. Experimental studies link TMAO exposure to arterial stiffness, endothelial dysfunction, macrophage polarization, vascular reactive oxygen species, and NLRP3 inflammasome activation (12, 13). Direct causal evidence in humans requires longitudinal and interventional confirmation.

Short-chain fatty acids (SCFAs). The major SCFAs (butyrate, propionate, and acetate) are produced through anaerobic fermentation of dietary fiber by colonic bacteria. SCFAs exert blood pressure effects through at least two receptor systems: olfactory receptor 78 (Olfr78), expressed in renal juxtaglomerular cells, promotes renin secretion in response to propionate and modestly elevates blood pressure, whereas G protein-coupled receptor 41 (GPR41), expressed in vascular smooth muscle cells, promotes vasodilation in response to acetate and propionate (14). The net effect depends on SCFA species, local concentrations, and receptor density. Critically, SCFA deficiency removes the butyrate-mediated anti-inflammatory and vascular protective effects, shifting the vascular milieu toward increased tone, oxidative stress, and endothelial inflammation.

Bile acids. Secondary bile acids generated by gut bacterial 7*α*-dehydroxylation (principally deoxycholic acid and lithocholic acid) signal through the farnesoid X receptor and Takeda G protein-coupled receptor 5 in vascular smooth muscle and endothelium. Dysbiosis-associated alterations in the bile acid pool promote vascular inflammation and impair endothelium-dependent vasodilation. The gut-liver-vascular bile acid axis represents an additional gut-to-blood pressure pathway.

Isovalerylcarnitine. A recent metabolomic study (14) identified isovalerylcarnitine as a novel gut microbiota-derived metabolite modulating salt-sensitive hypertension in mice through adrenal-renal mechanisms, illustrating that the metabolite repertoire linking gut dysbiosis to blood pressure extends well beyond TMAO and SCFAs (15, 16).

### 2.3 Gut barrier dysfunction and systemic inflammation

Gut dysbiosis promotes intestinal barrier dysfunction (“leaky gut”) through depletion of barrier-protective species (*Akkermansia muciniphila*, *Lactobacillus* spp.) that maintain mucus layer integrity and tight junction protein expression. The resulting increase in intestinal permeability facilitates translocation of lipopolysaccharide (LPS), a gram-negative bacterial outer membrane component, into the portal and systemic circulation. Circulating LPS activates Toll-like receptor 4 (TLR4) on vascular endothelial cells, smooth muscle cells, and macrophages, promoting NF-*κ*B-mediated transcription of pro-inflammatory cytokines (IL-6, TNF-α, IL-1β), endothelial dysfunction, and vascular remodeling (17).

Sustained low-grade endotoxemia may contribute to hypertension through multiple pathways: direct endothelial activation, sympathetic nervous system sensitization, and renal tubular dysfunction (18). The gut-immune-vascular circuit is detailed by Dinakis and Marques (19).

### 2.4 Autonomic and neurohumoral pathways

The gut communicates with cardiovascular control centers through vagal afferent fibers, enteric nervous system relays, and hypothalamic-brainstem circuits. Gut dysbiosis may impair gut-vagal afferent signaling, promote sympathetic predominance, and weaken parasympathetic modulation of heart rate and vascular tone (20). Reduced vagal tone may both follow and perpetuate hypertension, creating a bidirectional relationship in which dysbiosis is one possible upstream influence.

The renin-angiotensin system (RAS) is modulated by gut microbiota both locally through intestinal angiotensin-converting enzyme 2 (ACE2) expression and systemically. ACE2, a key RAS enzyme, is expressed in intestinal epithelium where it is regulated by microbial colonization (21). Dysbiosis alters intestinal ACE2 expression, potentially modifying systemic angiotensin II levels and blood pressure regulation. Indole-3 acetate, a gut microbial tryptophan metabolite, has been shown to limit dysbiosis-driven diastolic failure through hypothalamic orexin neuronal signaling (22), providing mechanistic evidence of gut metabolite → hypothalamic autonomic regulation → cardiovascular function.

### 2.5 Therapeutic modification of the gut-hypertension axis

Probiotic supplementation (predominantly *Lactobacillus* and *Bifidobacterium* species) reduces systolic blood pressure by approximately 3–4 mmHg in a meta-analysis of randomized controlled trials (23). This effect, while modest, is clinically meaningful in the context of population-level cardiovascular risk reduction. Prebiotics (non-digestible fiber substrates) improve blood pressure control by selectively increasing SCFA-producing bacteria (24). Fructooligosaccharides (FOS) have demonstrated direct endothelial function improvements in randomized controlled trial settings (25).

## 3 The gut microbiome in cognitive decline

### 3.1 Dysbiotic signatures in cognitive decline and dementia

Gut microbiome alterations in Alzheimer's disease and vascular cognitive impairment have now been systematically reviewed in multiple studies. A 2026 systematic review (26) confirmed associations between gut microbiota alterations and cognitive decline across human observational studies, with consistent findings including decreased *Bifidobacterium* and *Lactobacillus*, altered Firmicutes/Bacteroidetes ratio, and enrichment of pro-inflammatory gram-negative taxa (27, 28).

Comparing dysbiotic signatures in hypertension and AD reveals substantial overlap: both conditions share depletion of SCFA-producing bacteria (*Roseburia*, *Faecalibacterium prausnitzii*), reduced *Akkermansia muciniphila*, and enrichment of LPS-producing *Proteobacteria*. This overlap supports the hypothesis that a shared upstream dysbiotic state contributes to both conditions simultaneously, the conceptual basis of the double hit hypothesis. Plant-based dietary patterns and olive oil consumption, which favorably shift gut microbiota toward butyrate-producing taxa, are associated with improved cognitive trajectories (29, 30). Insulin resistance-mediated mechanisms may further link gut microbiota alterations to AD pathogenesis (31).

VCI-associated dysbiosis has been less extensively studied than AD-associated dysbiosis but shares features with both AD and hypertension-associated profiles, consistent with its mixed ischemic-degenerative pathogenesis.

The same directionality problem applies to cognitive cohorts. Cognitive impairment may change diet, mobility, gastrointestinal function, medication burden, and caregiver-dependent food choices, while age, cardiometabolic disease, renal function, socioeconomic conditions, and polypharmacy may influence both cognition and microbiome composition. In older adults, microbiome composition tracks diet, residence, frailty, comorbidity, and inflammatory status (79). These findings support association, not a presumption that dysbiosis preceded cognitive decline.

### 3.2 Metabolite-mediated neurodegeneration

TMAO and the brain. Elevated plasma TMAO is associated with worse cognitive performance, lower hippocampal volume, and higher amyloid burden in human cohort studies (32). In longitudinal Multi-Ethnic Study of Atherosclerosis data, higher TMAO levels were associated with faster cognitive decline after adjustment for measured covariates (33, 34); this association does not establish that TMAO is causal. Experimental studies have proposed several mechanisms, including altered blood-brain barrier (BBB) tight-junction integrity, microglial NLRP3 inflammasome activation, amyloid-*β* aggregation, and tau phosphorylation. Arangia and colleagues review TMAO as a candidate gut-brain-axis biomarker, but its clinical validity and utility remain unestablished (35). Dysbiotic taxa including *Bilophila wadsworthia* are associated with basal ganglia atrophy and structural cognitive impairment in neuroimaging studies (36). Dietary emulsifiers may contribute to gut dysbiosis-mediated cognitive decline through microbiome disruption and systemic inflammation (37).

Because vascular and neural associations have both been reported, TMAO is a candidate shared marker in the proposed framework, not an established mediator or validated integrated biomarker. Evidence that experimentally lowering TMAO prevents hypertension or cognitive decline in humans is lacking; dietary choline restriction, FMO3 inhibition, and other TMAO-directed strategies should therefore remain research hypotheses rather than clinical recommendations.

SCFA deficiency and the brain. Butyrate, the principal colonic SCFA, is a primary energy source for colonocytes and exerts anti-inflammatory effects through histone deacetylase inhibition. In the brain, butyrate crosses the BBB and suppresses microglial activation, promotes brain-derived neurotrophic factor expression, and supports oligodendrocyte maturation. SCFA deficiency removes this neuroprotective signal, permitting unchecked microglial activation and neuroinflammation. Connell and colleagues (38) review microbial metabolites as risk factors for age-related cognitive decline.

Bile acids. Gut-derived bile acids, substantially altered in dysbiosis, influence neurological function through multi-omics pathways linking deoxycholic acid and secondary bile acid species to neuroinflammation and cognitive impairment (39, 40).

Tryptophan metabolites. Microbial indole metabolites (indole-3 acetate, indole-3 propionic acid) modulate both gut and BBB permeability. *In vitro* evidence demonstrates that these metabolites regulate tight junction protein expression at the BBB in a dose-dependent manner (41, 42). Reduced production of protective indoles in dysbiosis may thus contribute to both gut leakiness and BBB vulnerability.

### 3.3 Neuroinflammatory pathways

LPS translocation from a leaky gut activates TLR4 on brain microglia and cerebral endothelial cells, triggering M1 microglial polarization with sustained production of IL-6, TNF-α, and IL-1β (43). Animal model evidence confirms that gut microbiota dysbiosis drives early AD pathogenesis through microglial activation (44, 45). Gut dysbiosis-derived metabolites promote neurodegeneration through multiple converging molecular cascades (46). Sustained microglial M1 activation impairs amyloid-*β* clearance by phagocytes, promotes tau hyperphosphorylation through kinase activation, and damages synaptic contacts through complement-mediated pruning (47). Microbiota-neuroimmune interactions regulate microglial function and neurological resilience across the life course (48).

Systemic inflammation originating from gut-derived LPS and bacterial metabolites creates a neuroinflammatory state that compromises neuronal resilience, reduces synaptic plasticity, and accelerates neurodegeneration independently of hypertension.

### 3.4 The vagus nerve as a gut-brain conduit

The vagus nerve provides a direct anatomical conduit between the enteric nervous system and brainstem nuclei, including the nucleus tractus solitarius, which projects to hippocampal and cortical networks involved in memory consolidation. Gut inflammation and dysbiosis impair normal vagal afferent signaling both through direct inflammatory damage to enteric neurons and through altered neuroactive metabolite production. Disrupted vagal tone thus simultaneously impairs hippocampal-dependent memory consolidation (cognitive consequence) and cardiovascular parasympathetic modulation (contributing to hypertension). This positions the vagus nerve as a convergence node in the gut-brain-vascular axis.

## 4 Hypertension-mediated cerebrovascular injury: the proposed indirect route

Evidence for the complete gut-hypertension-brain route remains limited. Its component associations are supported to different degrees, but no longitudinal human study has demonstrated the entire temporal sequence from dysbiosis through incident hypertension and cerebrovascular injury to cognitive decline. No study has directly tested an interaction between the two proposed routes. The subsections below therefore distinguish established mechanisms of hypertension-mediated brain injury from experimental or observational evidence that gut-derived signals may modify those mechanisms.

### 4.1 Hypertension, endothelial dysfunction, and cerebral small vessel disease

Cerebral small vessel disease (CSVD), including white matter hyperintensities (WMH), lacunar infarcts, cerebral microbleeds, and enlarged perivascular spaces, is a major structural pathway linking hypertension to cognitive impairment (49). Chronic hypertension exposes small vessels to mechanical stress, endothelial activation, lipohyalinosis, and impaired barrier function. CSVD burden is associated with deficits in processing speed and executive function, and hypertension and WMH burden are important correlates of cognitive impairment in CSVD cohorts (50, 51). Blood-pressure variability and elevated nighttime systolic pressure are also associated with adverse brain structure, neuroaxonal injury, and cognitive impairment (52–54).

Gut-related evidence is less direct. Circulating microbial products and metabolites can act on vascular endothelium, and TMAO has been associated with endothelial dysfunction and WMH burden (13, 34). Cross-sectional Framingham data associate specific taxa with magnetic resonance imaging measures of CSVD, and bacterial extracts from patients with arteriosclerotic CSVD induced retinoic acid receptor-related orphan receptor *γ*t-dependent IL-17A production in experimental systems (74, 75). Mendelian randomization and neuroimaging studies have also reported microbiome-related signals for CSVD outcomes (58, 59, 76). These designs strengthen the rationale for a gut contribution but do not demonstrate that dysbiosis causes hypertension and subsequently causes CSVD within the same individuals.

### 4.2 Cerebral autoregulation and neurovascular coupling

Cerebral autoregulation maintains cerebral blood flow across changes in systemic pressure. Chronic hypertension remodels resistance vessels and shifts the pressure range over which flow can be maintained, increasing vulnerability to hypoperfusion during blood-pressure reduction or variability. Hypertension also impairs endothelial signaling within the neurovascular unit, so neural activity may evoke an inadequate blood-flow response. Human evidence links altered cerebral blood-flow regulation in hypertension with cognitive performance (52), while experimental angiotensin II signaling can disrupt vasorelaxation in cerebral vessels (64). In a cross-sectional study of patients with CSVD, gut microbial differences correlated with imaging measures of neurovascular coupling and cognition (73); this association does not establish directionality.

Gut-derived metabolites, systemic inflammation, and autonomic imbalance could plausibly modify endothelial tone or neurovascular signaling, but the present evidence does not demonstrate this sequence directly. Human experimental endotoxemia has produced a more complex result, with lipopolysaccharide infusion enhancing rather than impairing dynamic cerebral autoregulation in healthy volunteers (81). Direct evidence that gut dysbiosis shifts the cerebral autoregulatory curve in humans is therefore insufficient. This link should be treated as a testable mechanism rather than an established step.

### 4.3 Blood-brain barrier and glymphatic function

Hypertension can activate cerebral endothelial cells and pericytes, increase matrix metalloproteinase activity, degrade tight-junction proteins, and increase blood-brain barrier permeability (11, 55). A disrupted BBB can expose the parenchyma to circulating inflammatory mediators. In spontaneously hypertensive stroke-prone rats, fecal microbiota manipulation implicated the gut microbiome in BBB disruption, providing experimental evidence for a gut-to-barrier effect in a hypertensive background (62). This animal result does not establish the same pathway in humans.

Glymphatic dysfunction may further impair clearance of amyloid-*β* and tau in neurovascular disease (56). A cross-sectional imaging study in ulcerative colitis associated lower along-the-perivascular-space (ALPS) indices with inflammation and SCFA-related taxa, providing gut-disease evidence outside a hypertension population (77). Direct evidence that gut dysbiosis impairs glymphatic clearance in people with hypertension is presently insufficient. The proposed connection rests on shared endothelial, inflammatory, and barrier pathways and remains hypothesis-generating.

### 4.4 A testable double-hit hypothesis

The model posits two mechanistically distinguishable routes that may occur together and may interact, but their temporal concurrence and statistical interaction have not been established (Figure 1).

**Figure 1:**
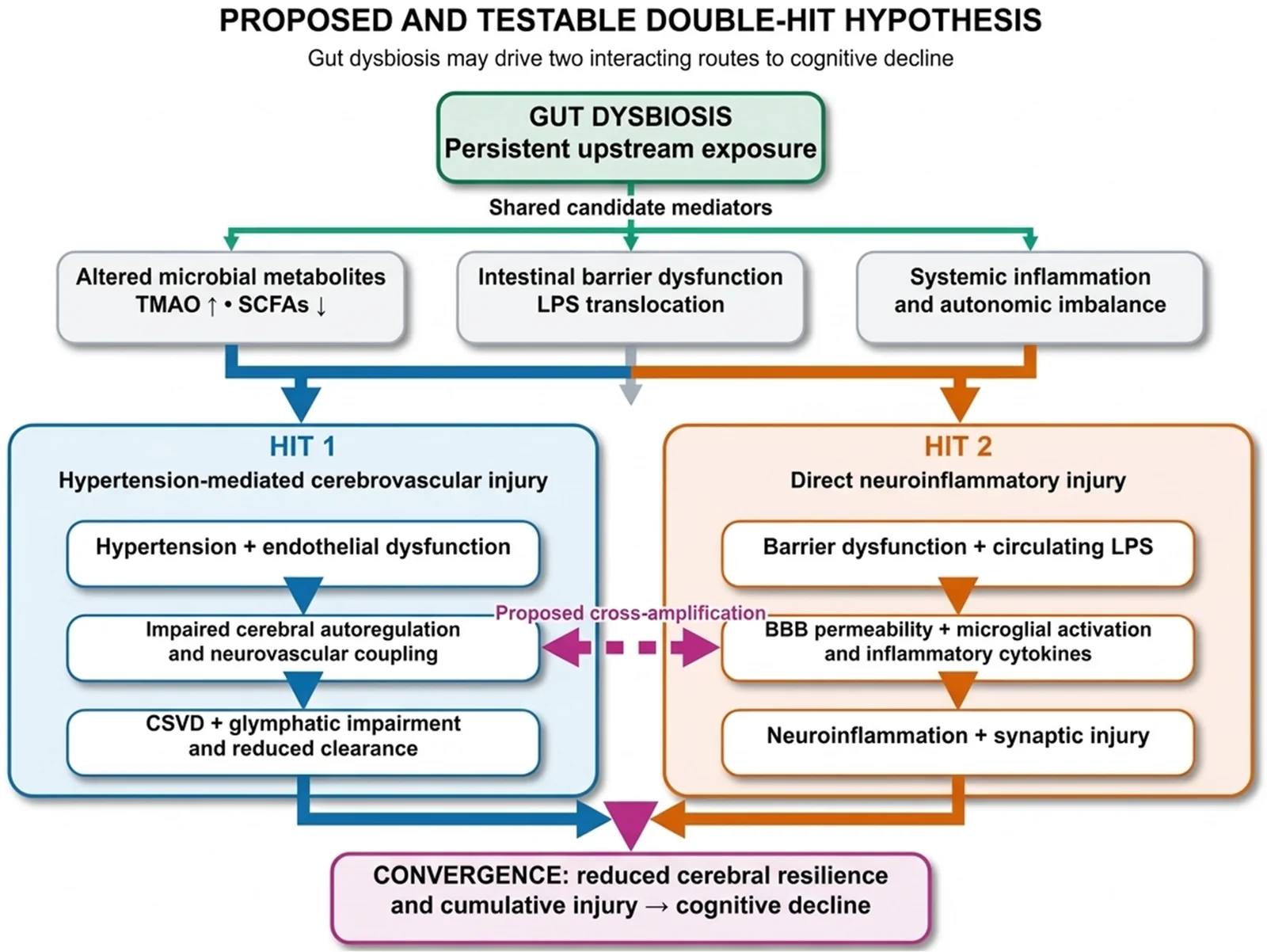
Proposed double-hit model of the gut-brain-vascular axis. Gut dysbiosis is positioned upstream of shared candidate signals, including intestinal barrier dysfunction and lipopolysaccharide exposure, TMAO, SCFA deficiency, immune activation, and autonomic or renin-angiotensin signaling. The proposed hypertension-mediated route proceeds through endothelial dysfunction, cerebral small vessel disease, impaired cerebral autoregulation and neurovascular coupling, and BBB or glymphatic dysfunction. The proposed direct route proceeds through BBB effects, microglial activation, and neuroinflammation. Both routes converge on cognitive decline. Solid arrows denote component links with direct experimental, longitudinal, or established clinical support; dashed arrows denote indirect or proposed links. Interaction between the routes is hypothesized and has not been demonstrated statistically. BBB, blood-brain barrier; CSVD, cerebral small vessel disease; LPS, lipopolysaccharide; RAS, renin-angiotensin system; SCFA, short-chain fatty acid; TMAO, trimethylamine N-oxide.

Route 1, hypertension-mediated cerebrovascular injury. Dysbiosis-associated metabolites, immune activation, autonomic imbalance, and endothelial dysfunction may contribute to hypertension. Hypertension can then promote CSVD, impaired cerebral autoregulation, neurovascular uncoupling, BBB dysfunction, and cerebral hypoperfusion.

Route 2, direct neuroinflammatory signaling. Intestinal barrier dysfunction and altered microbial signals may contribute to systemic inflammation, BBB effects, microglial activation, and neuroinflammatory injury without hypertension as a necessary intermediate.

A central, testable prediction is that coexisting hypertension-mediated cerebrovascular vulnerability and gut-associated neuroinflammatory signaling produce greater cognitive injury than either route alone. Testing this prediction requires longitudinal studies with prespecified interaction terms or factorial or interventional designs; the current literature does not provide such a test.

### 4.5 Evidence spanning the three domains

Direct human evidence spanning microbiome, hypertension, and cognition is sparse. A cross-sectional study reported correlations among gut microbiota, intestinal permeability, and cognitive impairment in older adults with hypertension (57). Mendelian randomization and neuroimaging reports address microbiome-related exposures and CSVD outcomes, but they do not observe the full temporal sequence (58, 59). Population and metagenomic studies connect gut profiles with cardiometabolic or stroke-related outcomes, again without demonstrating the complete pathway (60, 61).

Experimental work provides stronger evidence for individual links. Fecal microbiota transfer in hypertensive rats implicated the microbiome in BBB disruption (62), and an exploratory human FMT study examined microbiome modification in hypertension (63). Neither study tested the proposed interaction on cognitive outcomes.

Faraco reviewed a dietary salt-gut dysbiosis-vascular dysfunction-cognitive impairment pathway, and Pala and colleagues reviewed microbiota as a modifier of hypertension-mediated organ damage (5, 6). The present model therefore builds on, rather than claims priority over, those syntheses. Its narrower contribution is to place a direct neuroinflammatory route beside a specifically hypertension-mediated cerebrovascular route and to identify their interaction as an untested prediction.

## 5 Shared candidate mechanisms

The mechanisms detailed in Sections 2 and 3 converge at a smaller set of candidate nodes. Rather than restating each pathway, Table 1 summarizes how the same signal could affect vascular and neural compartments.

**Table 1:** Candidate mechanistic nodes linking gut microbiome-related signaling to vascular and neural outcomes.

Candidate node	Vascular relevance	Neural relevance	Evidence boundary
TMAO	Experimental evidence links TMAO to endothelial dysfunction and arterial stiffness; human WMH associations have been reported (13, 34)	Associated with cognitive outcomes; BBB or microglial effects remain mechanistic proposals (32–35)	Human evidence is principally observational; causal effects across the full pathway are unproven.
SCFA deficiency	Loss of anti-inflammatory and endothelial-protective signaling	Loss of proposed microglial, epigenetic, and neurotrophic support (31, 38)	Mechanistic evidence exceeds direct clinical evidence.
Intestinal barrier dysfunction and LPS	Systemic immune and endothelial activation (17–19)	BBB exposure and microglial or astrocytic activation (43, 48)	Directionality and exposure measurement remain inconsistent in human cohorts.
Autonomic and renin-angiotensin signaling	Sympathovagal imbalance and blood-pressure regulation (20–22)	Cerebral vascular tone and neuroimmune signaling (55, 64)	The gut-to-cerebral sequence has not been demonstrated prospectively.
BBB dysfunction	Target-organ injury in hypertension (11, 62)	Entry of circulating inflammatory mediators and reduced neural resilience	BBB disruption is a convergence site, not proof of interaction between routes.

These nodes provide biological plausibility and measurable targets, but they do not establish that the two routes operate concurrently or synergistically in humans.

## 6 Therapeutic implications

The proposed framework motivates testing whether microbiome-directed interventions can affect blood pressure and cognitive outcomes together, but no intervention has demonstrated simultaneous benefit across the microbiome, blood-pressure, and cognitive domains. The evidence in each domain therefore requires separate appraisal. Table 2 compares dietary patterns by outcome and study design, and Table 3 summarizes therapeutic evidence without inferring cross-domain mediation.

**Table 2:** Dietary patterns: outcome-specific evidence and evidentiary boundaries.

Diet	Blood-pressure evidence	Cognitive evidence	Microbiome evidence	Cross-domain boundary
DASH	Randomized trials and meta-analyses support BP reduction; magnitude varies by population and sodium intake.	Limited and not definitive.	Early clinical evidence; a microbiome-focused randomized trial has been registered (65).	BP benefit is established independently of microbiome or cognitive mediation.
Mediterranean	Clinical trials and meta-analyses support modest BP reduction.	Prospective cohorts report better cognitive outcomes (30, 66); a combined diet-activity pilot trial is registered (67).	Human studies report compositional differences and butyrate-producing taxa (66).	The three outcomes are generally derived from different studies; microbiome mediation is unproven.
MIND	Direct BP trial evidence is limited.	Prospective cohorts associate adherence with slower cognitive decline (28, 29).	A review proposes microbiota as a possible mediator (68).	Neither causal microbiome mediation nor a combined BP-cognition benefit has been established.
High-salt/Western	Trials and epidemiologic studies support adverse BP effects.	Cognitive findings are primarily observational.	Experimental and observational studies report reduced *Lactobacillus* and other compositional changes (8).	Parallel adverse findings do not demonstrate a single causal sequence in humans.

*BP, blood pressure.*

**Table 3:** Therapeutic interventions: domain-specific evidence and cross-domain gaps.

Intervention	Blood-pressure evidence	Cognitive evidence	Microbiome evidence	Cross-domain inference
Probiotics (*Lactobacillus* spp.)	Meta-analysis of RCTs reports a modest SBP reduction (23).	Human randomized evidence is limited (69).	Target engagement is expected and strain-specific.	No trial has tested BP and cognition as co-primary outcomes.
Prebiotics (inulin, FOS)	Clinical studies report BP or endothelial effects (24, 25).	Evidence is predominantly preclinical.	Clinical studies report increases in SCFA-producing taxa.	Cognitive benefit cannot be inferred from BP or microbiome changes.
Mediterranean diet	Clinical evidence supports modest BP reduction.	Prospective evidence supports selected cognitive outcomes (30, 66); a combined diet-activity pilot trial is registered (67).	Human studies report compositional changes (66).	Evidence comes from heterogeneous studies; mediation is unproven.
MIND diet	Direct BP evidence is limited.	Prospective observational evidence supports slower cognitive decline (28, 29).	A review proposes possible microbiome mediation (68).	Causal microbiome mediation has not been established.
DASH diet	Randomized evidence supports BP reduction.	Evidence is limited.	Microbiome-specific clinical evidence remains early (65).	Established BP benefit does not establish microbiome or cognitive benefit.
FMT	Causal BP evidence is preclinical; one human pilot assessed feasibility (7, 62, 63).	Preclinical only.	Directly changes microbial composition.	No human trial has measured BP and cognitive outcomes together.
Butyrate/postbiotics	Vascular evidence is primarily preclinical.	Neuroprotection evidence is primarily preclinical.	Acts through microbial products rather than community replacement.	Human clinical benefit remains untested.
ACE inhibitors/ARBs	Established antihypertensive therapy.	Any cognitive effect is attributed to BP control, not microbiome modulation.	Clinically consequential microbiome effects are unproven.	Microbiome mediation should not be inferred.
Glucagon-like peptide-1 receptor agonists/sodium-glucose cotransporter-2 inhibitors	Established metabolic and cardiorenal benefits; BP effects vary.	Cognitive evidence is emerging.	Treatment-associated compositional changes have been reported (70).	Microbiome changes may be secondary; mediation has not been demonstrated.

Evidence within each outcome domain derives from different study designs and populations. Findings in one domain should not be interpreted as evidence that microbiome change mediates blood-pressure or cognitive effects unless mediation or intervention studies directly demonstrate that pathway.

ACE, angiotensin-converting enzyme; ARB, angiotensin II receptor blocker; BP, blood pressure; FMT, fecal microbiota transplantation; FOS, fructooligosaccharides; MIND, Mediterranean-DASH Intervention for Neurodegenerative Delay; RCT, randomized controlled trial; SBP, systolic blood pressure; SCFA, short-chain fatty acid.

### 6.1 Dietary interventions

DASH (Dietary Approaches to Stop Hypertension) diet. Randomized trials and meta-analyses support blood-pressure reduction with DASH, although the magnitude varies with baseline blood pressure, sodium intake, and trial design. Its fiber content provides a plausible substrate for SCFA-producing bacteria, but direct microbiome evidence is less mature; a recent randomized trial protocol was designed specifically to examine this question (65). Evidence for a direct cognitive benefit is also limited. The established blood-pressure effect should not be interpreted as evidence that microbiome changes mediate cardiovascular or cognitive outcomes.

Mediterranean diet. Clinical studies support cardiovascular benefit and modest blood-pressure reduction, while prospective cohorts associate Mediterranean-pattern adherence with cognitive outcomes (30, 66). Microbiome studies report differences in diversity and butyrate-producing taxa, but these findings do not establish mediation. A 2026 pilot-trial protocol will test a combined Mediterranean-diet and physical-activity intervention; because the interventions are combined, any eventual effect could not be attributed to diet or microbiome change alone (67).

Mediterranean-DASH Intervention for Neurodegenerative Delay (MIND) diet. The MIND diet is associated with slower cognitive decline in prospective dietary studies (28, 29). A recent review proposed gut microbiota as a possible mediator of this association (68), but the available evidence does not establish microbiome mediation. Direct blood-pressure trial evidence is limited.

### 6.2 Probiotics and prebiotics

Probiotics (blood pressure). Meta-analytic evidence from randomized controlled trials (RCTs) supports a modest but significant reduction in systolic blood pressure (approximately 3–4 mmHg) with probiotic supplementation using *Lactobacillus* and *Bifidobacterium* strains (23). The antihypertensive mechanism involves SCFA production, ACE inhibitory peptide release, and anti-inflammatory effects.

Probiotics (Cognition). “Psychobiotics,” probiotic strains with proposed neuroactive properties, have reduced neuroinflammatory markers and improved cognitive performance in animal models. Human randomized-trial evidence remains limited (69). No published probiotic trial has assessed blood pressure and cognition as co-primary outcomes.

Prebiotics (blood pressure). Prebiotic fiber supplementation increases SCFA-producing bacteria and improves blood pressure control (24). Inulin and fructooligosaccharides (FOS) are the most studied prebiotic substrates for blood-pressure modification, with RCT evidence demonstrating endothelial function improvements (25).

### 6.3 Postbiotics

Postbiotics, defined as preparations of inanimate microorganisms or their components that confer health benefits, include SCFA preparations, indole derivatives, and microbial cell-wall components. Butyrate supplementation (as sodium butyrate or tributyrin) reduces neuroinflammation and improves cognitive outcomes in rodent models of AD and VCI through histone deacetylase inhibition and microglial homeostasis restoration. Tryptophan-derived indoles (indole-3 acetate, indole-3 propionic acid) modulate BBB permeability and may reduce both hypertensive endothelial activation and neuroinflammatory BBB disruption (22, 42).

### 6.4 Fecal microbiota transplantation

FMT provides proof-of-concept for gut dysbiosis → BP causality in animal models (7, 62). Human FMT for blood-pressure management has entered early-stage clinical investigation; an exploratory trial demonstrated feasibility and preliminary microbiome modification in patients with hypertension (63). A trial of FMT from healthy donors to patients with hypertension, with concurrent cognitive monitoring, would directly test the gut-brain-vascular axis hypothesis in humans.

### 6.5 Antihypertensive medications and the microbiome

Antihypertensive therapy has established benefits through blood-pressure lowering, but evidence that angiotensin-converting enzyme inhibitors, angiotensin II receptor blockers, or *β*-blockers produce clinically consequential microbiome changes is insufficient. Reviews also describe gut-microbiome changes during treatment with glucagon-like peptide-1 receptor agonists (GLP-1RAs) and sodium-glucose cotransporter-2 inhibitors (SGLT2 inhibitors) (70), but these observations do not show that microbiome modulation mediates their cardiovascular or cognitive effects. Changes in metabolic status, blood pressure, renal function, diet, concomitant medication use, or gut physiology may instead account for the observed microbial differences. The SPRINT-MIND trial found that intensive blood-pressure lowering reduced the risk of mild cognitive impairment (71); it did not test microbiome mediation (2).

## 7 Conclusion

This review proposes the gut-brain-vascular axis as a conceptual model in which dysbiosis may contribute to cognitive decline through direct neuroinflammatory signaling and an indirect route involving hypertension-mediated cerebrovascular injury. Hypertension is the specific vascular intermediate in the model; broader vascular disease is outside its primary scope.

The double-hit hypothesis is potentially informative for study design, but the proposed interaction remains untested. TMAO, SCFA deficiency, intestinal barrier dysfunction, autonomic signaling, and systemic inflammation are candidate shared nodes that can be measured across the two routes.

Dietary interventions, probiotics, prebiotics, and postbiotics have been studied within individual microbiome, blood-pressure, or cognitive domains, but integrated trials are lacking. Clinical claims should therefore remain tied to the outcome directly measured by each study.

Prospective studies that measure microbiome composition (82), ambulatory blood pressure, cerebrovascular function, and cognition in the same participants are needed to determine whether the proposed pathways occur sequentially, independently, or interactively.
